# Transmissibility of intra-host hepatitis C virus variants

**DOI:** 10.1186/s12864-017-4267-4

**Published:** 2017-12-06

**Authors:** David S. Campo, June Zhang, Sumathi Ramachandran, Yury Khudyakov

**Affiliations:** 1 Division of Viral Hepatitis, Molecular Epidemiology and Bioinformatics, Centers for Disease Control and Prevention, Atlanta, GA USA; 2 Department of Electrical Engineering, University of Hawaii, Manoa, HI USA

## Abstract

**Background:**

Intra-host hepatitis C virus (HCV) populations are genetically heterogeneous and organized in subpopulations. With the exception of blood transfusions, transmission of HCV occurs via a small number of genetic variants, the effect of which is frequently described as a bottleneck. Stochasticity of transmission associated with the bottleneck is usually used to explain genetic differences among HCV populations identified in the source and recipient cases, which may be further exacerbated by intra-host HCV evolution and differential biological capacity of HCV variants to successfully establish a population in a new host.

**Results:**

Transmissibility was formulated as a property that can be measured from experimental Ultra-Deep Sequencing (UDS) data. The UDS data were obtained from one large hepatitis C outbreak involving an epidemiologically defined source and 18 recipient cases. *k*-Step networks of HCV variants were constructed and used to identify a potential association between transmissibility and network centrality of individual HCV variants from the source. An additional dataset obtained from nine other HCV outbreaks with known directionality of transmission was used for validation.

Transmissibility was not found to be dependent on high frequency of variants in the source, supporting the earlier observations of transmission of minority variants. Among all tested measures of centrality, the highest correlation of transmissibility was found with Hamming centrality (*r* = 0.720; *p* = 1.57 E-71). Correlation between genetic distances and differences in transmissibility among HCV variants from the source was found to be 0.3276 (Mantel Test, *p* = 9.99 E-5), indicating association between genetic proximity and transmissibility. A strong correlation ranging from 0.565–0.947 was observed between Hamming centrality and transmissibility in 7 of the 9 additional transmission clusters (*p* < 0.05).

**Conclusions:**

Transmission is not an exclusively stochastic process. Transmissibility, as formally measured in this study, is associated with certain biological properties that also define location of variants in the genetic space occupied by the HCV strain from the source. The measure may also be applicable to other highly heterogeneous viruses. Besides improving accuracy of outbreak investigations, this finding helps with the understanding of molecular mechanisms contributing to establishment of chronic HCV infection.

## Background

Hepatitis C virus (HCV) infects nearly 3% of the world’s population and is a major cause of liver disease worldwide [1]. HCV infection is an important US public health problem, being the most common chronic blood-borne infection and the leading cause for liver transplantation [2]. Since 2007, HCV has surpassed Human Immunodeficiency Virus (HIV) as a cause of death in the United States [3]. It is estimated that 2.7–3.9 million people in the United States have chronic HCV infection and that > 15,000 die each year from HCV-related disease, with mortality expected to rise in the coming years [4]. Approximately 80% of patients who become infected with HCV develop chronic infections and are at risk for advanced liver disease; 15%–30% of these patients progress to liver fibrosis and cirrhosis and up to 5% die from liver failure due to cirrhosis or hepatocellular carcinoma [2]. Outbreaks of HCV infections are associated with unsafe injection practices, drug diversion, and other exposures to blood and blood products [5].

RNA viruses such as HCV exist as a heterogeneous population of closely related but genetically distinct variants in each infected person [6, 7]. During a transmission event, this variability creates an opportunity for differences in the genetic composition of variants found in the source and recipients. For HIV, it is known that, although source may have many viral variants, recipients are productively infected by a small number of them, often only one [8]. For HCV, several studies have shown that only a small number of HCV variants establish infection in a new host [9–12], commonly resulting in a profound founder effect [13].

Establishment of new infections originating from a small, random sample of intra-host variants from the source (often referred to as a bottleneck) may explain the genetic differences in viral populations found between the source and recipients. A fact that does not support this simple explanation is that a transmission/founder (T/F) virus is often different from major variants present in the source [8]. It is also important to note that due to a high rate of mutations, the viral population (often sampled weeks or months after the transmission event) may be greatly different from the population existing in the source at the time of transmission. Thus, there are a variety of transmission events that can contribute to detected genetic differences between viral populations identified in the source and recipients at the time of sampling.

Besides stochasticity of transmission, changes in genetic composition of the source and recipient populations may be explained by differences in biological properties among intra-host viral variants. It is conceivable that viral variants may differ in their capacity to be transmitted and to establish the first detectable viral population in a new host, with some being more likely to be T/F variants. Viral envelope glycoproteins, which are involved principally in virus attachment and entry into target cells, are likely to correlate with such transmission-related properties. Several recent HIV studies have given support to this active selection model by showing that newly acquired variants often have shorter glycosyl residues and/or less glycosylated envelope glycoproteins than those present in chronically infected persons [8]. For HCV, there are two main lines of evidence for the active selection model. First, Kell et al. [14] showed that T/F variants are recognized by retinoic acid-inducible gene I (RIG-I) in a manner dependent on length of the U-core motif of the poly-U/UC pathogen-associated molecular pattern (PAMP) and are recognized by RIG-I to induce innate immune responses that restrict acute infection. Second, as we have previously shown, physicochemical properties within the hypervariable region 1 (HVR1) of E2 envelope glycoprotein are significantly different between HCV variants found in acutely and chronically infected individuals [15]. This finding was recently confirmed in a study of three transmission pairs that found a common HVR1 amino acids pattern in transmitted HCV variants, with each transmission pair sharing specific patterns [11].

Finding specific phenotypic properties contributing to transmissibility of variants can help to understand the establishment of HCV chronic infection, improve accuracy of outbreak detection, and rationally design strategies for preventing HCV infection by limiting the search space for vaccine candidates. Here, we define transmissibility as a property that can be measured from experimental data obtained using ultra-deep sequencing (UDS) and identify properties of the source HCV variants that are associated with transmissibility.

## Methods

### Data

We conducted an in-depth analysis of one HCV infection outbreak (hereinafter referred to as the AW outbreak) where the source case of HCV infection was known. The AW outbreak investigation started with identification of two patients diagnosed with acute HCV infection from the same hospital. Further investigation implicated a drug-diverting; HCV-infected surgical technician as the source of the outbreak. Sera from the source and patients found to be serologically HCV positive were used to conduct HCV sequence analysis. In total, 5970 patients were notified of their possible exposure to HCV, 88% of whom were tested and had results reported to the state public health department. Ultimately, 18 patients had HCV sequences highly related to the surgical technician’s virus [16]. The associations identified from the AW outbreak analysis were further tested using data from nine other transmission clusters with known sources identified from eight additional outbreak investigations [16–22]. All nine outbreaks we examined were serologically confirmed, epidemiologically defined, and reported to the Centers for Disease Control and Prevention between 2008-2013 [5].

### Sequencing

The HCV strain identified in the AW outbreak was sequenced from all 19 cases using UDS. PCR products were pooled and subjected to pyrosequencing using GS FLX Titanium Sequencing Kit (454 Life Sciences, Roche, Branford, CT). The UDS files were processed using the error correction algorithms KEC and ET [23]. All sequence variants represented with a single UDS read were excluded from consideration. No sequences were obtained from one recipient despite several attempts. It must be noted that UDS was used only in the AW outbreak. Sequences for the other outbreaks were obtained using the End-Point Limiting-Dilution Real-Time PCR method for sequencing of multiple HVR1 clones [24–26]. For each sample of HCV sequences, a Multiple Sequence Alignment (MSA) was created using MAFFT 7.221 [27]. The primer sequences were removed and the final sequences were 264 nucleotides in length.

### Transmissibility

We developed a new measure of transmissibility for each variant in a source. Let *R* denote a set of recipients, with N being a total number of recipients. Let *R*_*j*_ be a set of distinct variants in recipient *j*. A total number of unique variants in recipient *j* is |*R*_*j*_|. For each variant *i* in the source, the average distance to recipient *R*_*j*_ is:

$$ d\left(i,{R}_j\right)=\frac{1}{\mid {R}_j\mid }{\sum}_{k=1}^{\mid {R}_j\mid }d\left(i,k\right), $$

where *d*(*i*, *k*) is the Hamming distance between a source’s variant *i* and recipient’s variant *k*. Rather than using a raw value *d*(*i*, *R*_*j*_), we used the rank of each source variant with respect to recipient:

$$ r\left(i,{R}_j\right)=\mathit{\operatorname{rank}}\left(-d\left(i,{R}_j\right)\right) $$

**Fig. 1:**
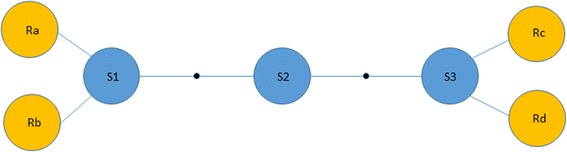
Diagram illustrating multiple clusters of transmissibility. There are three variants in the source (S1, S2 and S3). The lines represent one-step mutation, showing that S1 is very close to recipients Ra and Rb, whereas S3 is very close to recipients Rc and Rd. If we use the arithmetic mean of the genetic distances, all three sequences would get the same value (3), which would assign an artificially low value to S2. If we use the harmonic mean instead, S1 and S3 have a smaller value than S2 (1.5 vs 2)

Finally, transmissivity *T*_*i*_ of each variant is the harmonic mean of its rank with respect to all recipients (Fig. 1):

$$ {T}_i=\frac{N}{\sum_{j=1}^{\mid R\mid}\frac{1}{r\left(i,{R}_j\right)}} $$

We considered several transformation schemes such as the top quartile: for each recipient, the sequence was marked as 1 if it belongs to the top quartile of genetic distances, or 0 if otherwise, which highly correlated with the rank-based measure (*r* = 0.8625; *p* value = 5.82 E-132). However, we found ranking more suitable because, in the case of one or few recipients, the top quartile did not generate a continuous value for all sequences;

### Networks

For the set of 6231 distinct variants found in the AW outbreak, we visualized the sequence similarity by means of a k-step network as previously described [7, 24, 28]; nodes in the k-step network correspond to variants. The k-step network is equivalent to the union of all possible Minimum Spanning Trees and allows one to efficiently visualize the genetic relatedness among all variants present in a sample. The networks were drawn using GEPHI software [29].

### Variant properties

The read frequency of a variant is the number of cleaned experimental reads associated with that particular sequence from experiments. We previously showed that experimentally obtained frequencies are highly correlated with true proportions found in the sample [23]. Several topological properties of the k-step network were measured for each node: 1) degree centrality (number of links), 2) closeness centrality (reciprocal of the average shortest path) [30], 3) betweenness centrality (fraction of shortest path that goes through each node) [31], and 4) k-shell (a degree-based decomposition) [32]. Finally, the Hamming centrality of each sequence was calculated. Hamming centrality is analogous to closeness centrality [30] but it uses the matrix of Hamming distances among all pairs of sequences instead of the matrix of shortest paths in the network. Let N be the number of variants in a sample, the Hamming centrality (*HC*) of variant *x* is given by:

$$ {HC}_x=\frac{1}{\sum_yd\left(y,x\right)N-1} $$

where *d(y, x)* is the hamming distance between variants *x* and *y*.

### Hypothesis testing

We performed a Mann-Whitney U test to compare the frequency distributions of shared and non-shared variants. In order to evaluate if location in sequence space was associated with Transmissibility, we performed a Mantel test [33]. The Mantel test is a statistical permutation test (*n* = 10,000) of the correlation between two dissimilarity matrices: the first matrix is the Hamming distance between any two variants, whereas the second matrix is the absolute difference in their transmissibility.

## Results

### Frequency of shared HCV variants

**Fig. 2:**
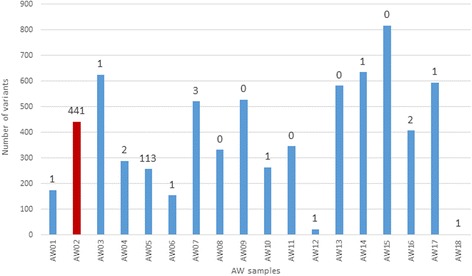
Number of variants in each sample of the AW outbreak. The number above each bar indicates the number of HCV variants that are shared with the source (AW2, shown in red)

The data available from the AW outbreak were especially amenable to analysis of transmissibility of intra-host HCV variants: (1) an epidemiologically established transmission from a single source case to 18 recipients over a 5-week period [22]; and (2) a large number of intra-host HCV HVR1 variants sampled from each case using UDS [23]. Sequences were obtained from the source and all but one recipient. A total of 6231 HCV variants obtained from the outbreak cases were represented by 137,691 sequence reads. An average of 388.16 variants, ranging from 2 to 816, were sampled from each case (Fig. 2). The confirmed outbreak source is designated as AW02. Although the number of variants sampled from the source was not highest among all cases, the intra-host HCV population had the highest level of nucleotide diversity [24]. Of the 441 unique source variants, 119 were shared with the recipients. Out of the 17 recipients, 12 shared at least one variant with the source (Fig. 2). While most recipients had only a few variants in common with the source, patient AW05 shared 113.

HCV variant frequencies, defined by the number of sequence reads representing each variant, varied in a broad range. Shared variants had a 6.4 times higher average frequency than non-shared variants (43.23 vs 6.70, *p* value <0.0001). Despite this observation, correlation between the number of recipients, where a certain source variant was found, and its frequency in the source was very low (*r* = 0.0949; *p* = 0.0464). This correlation, however, improved when Log10 of frequency was used (*r* = 0.4020; *p* < 0.0001). In support of this finding, the most frequent source variant (35.30% of the whole population) was found in only one of the 17 recipients, whereas the most shared variant, found in 8 recipients, was represented in the source by only a few sequence reads (0.11% of the whole population, less than 24.71% of all source variants).

### Genetic relatedness of HCV variants

**Fig. 3:**
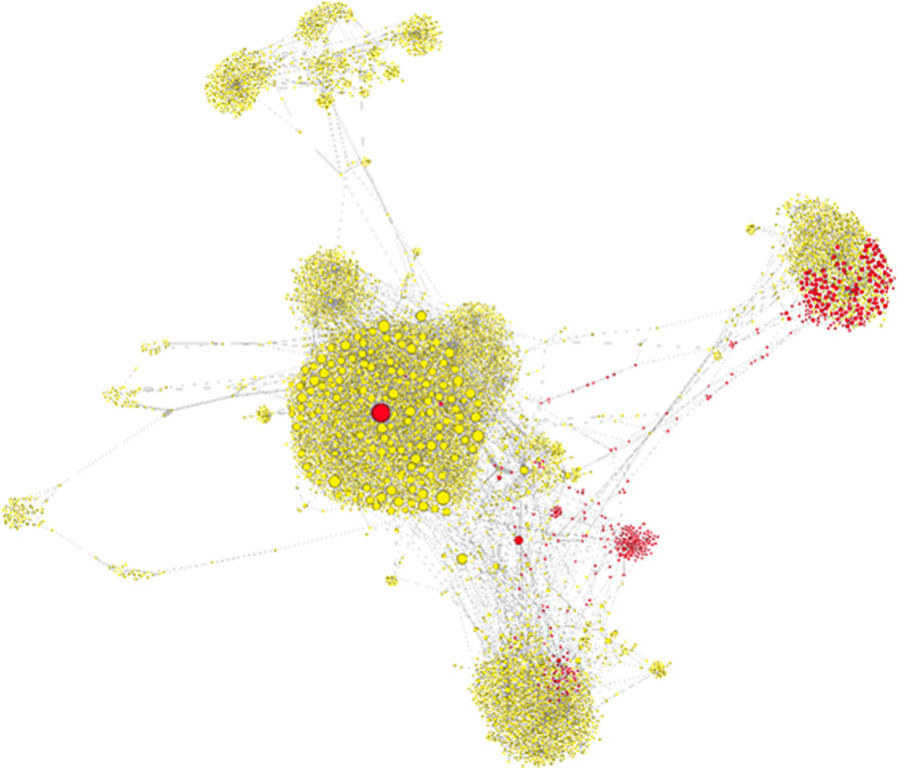
k-step network of the AW outbreak. Each node is a distinct HCV variant (*n* = 6231) found among the 18 samples. The size of the node is proportional to the number of samples that share that variant. Red nodes are variants found in the source; yellow nodes are variants found exclusively among recipients. Out of 11,418 links, 94.35% have a Hamming distance of 1, 5.24% a distance of 2, 0.32% a distance of 3, 0.06% a distance of 4, and 0.03% a distance of 15

The genetic relationships among HCV variants from the source and recipients was visualized using a k-step network, where each node is a distinct HCV variant (*n* = 6231) and the size of the node is proportional to the number of samples that share that variant (Fig. 3). There are several modules in the network, each representing a cluster of closely related HCV variants. The source variants are distributed among several clusters. The cluster containing a majority of HCV variants that were common among recipients was built around a single source variant. This particular source variant was shared with eight recipients, indicating a variable capacity of different source variants to serve as founders in these recipients.

### Transmissibility

**Fig. 4:**
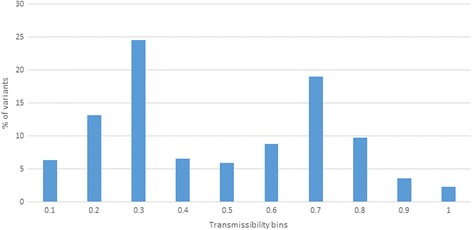
Frequency distribution of transmissibility. Percentage of variants in the source found in each transmissibility category

Transmissibility measured using intra-host variants shared between source and recipients is highly sensitive to stochastic disparities associated with variant sampling and variation in evolutionary history in infected persons. The transmissibility measure developed here reduces this sensitivity. A bimodal frequency distribution of transmissibility values for HCV variants (Fig. 4) reflects a complex modular organization of intra-host HCV population in the source (Fig. 3). This suggests the existence of more than one cluster, from which founders of the acute populations in recipients were recruited, with only very few variants having the highest values of transmissibility.

**Fig. 5:**
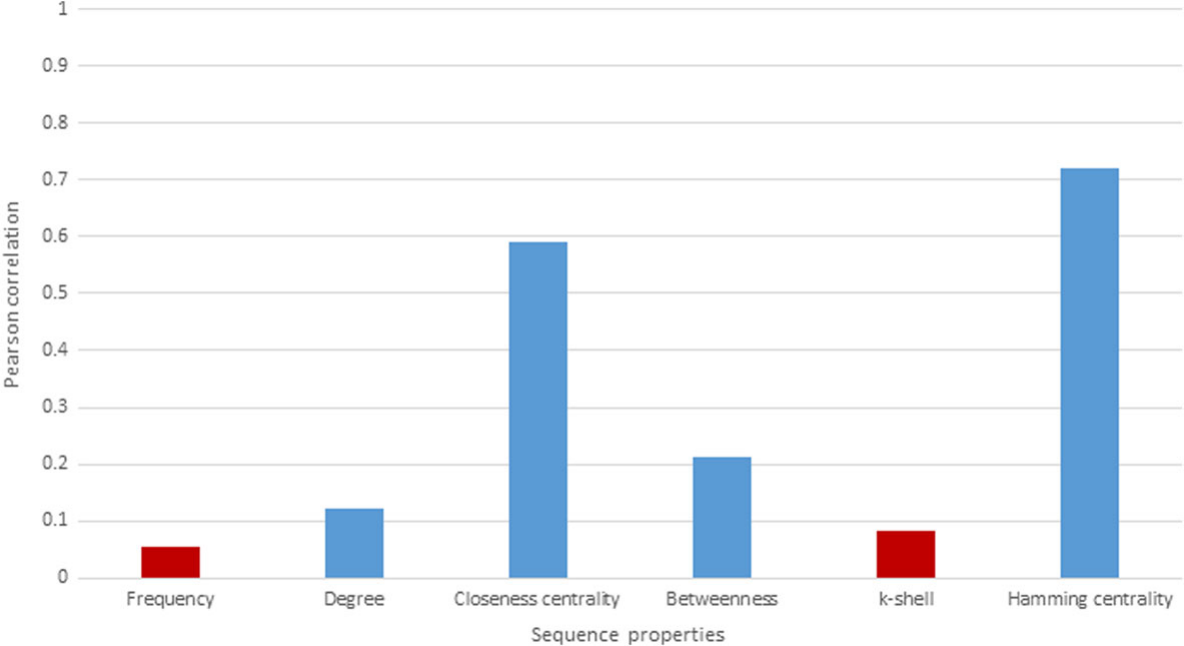
Pearson correlation between transmissibility and different sequence properties. Blue bars correspond to *p*-values lower than 0.05, whereas red bars correspond to p-values higher than > 0.05

**Fig. 6:**
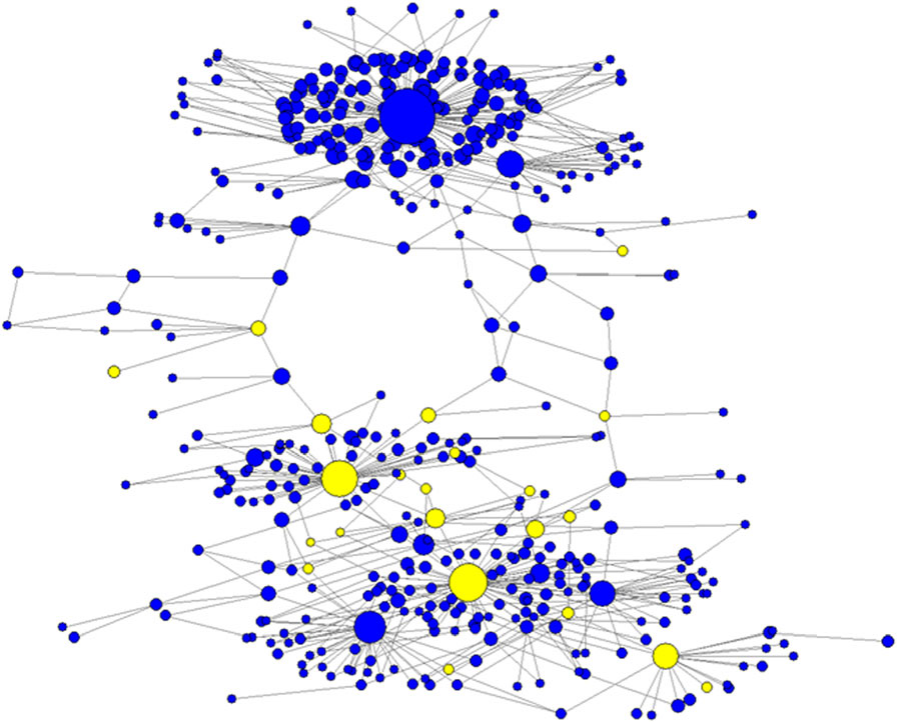
k-step network of the variants found in the source. Yellow nodes represent variants with a transmissibility value in the top 5%. The size of the node is proportional to the square root of the variant frequency

Pearson correlation was calculated between several sequence properties and transmissibility, though variant frequency did not correlate with transmissibility (Fig. 5). The source variants with high transmissibility had a moderate frequency (Fig. 6). Transmissibility was found to correlate with several measures of network centrality, including degree, closeness, and betweenness centrality. However, the highest correlation was found with Hamming centrality (*r* = 0.720; *p* = 1.57 E-71), which is calculated from the matrix of Hamming distances rather than from the k-step network.

**Fig. 7:**
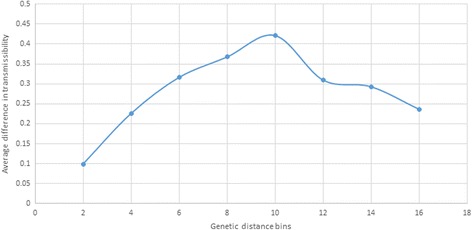
Transmissibility and genetic distance. Average difference in transmissibility over increasing genetic distance categories

We checked whether genetically close sequences have similar values of transmissibility. Correlation between matrices of genetic distances and differences in transmissibility among HCV variants was found to be low but significant (*r* = 0.3276; Mantel Test *p* value = 9.99 E-5). Figure 7 shows how the average difference in transmissibility increases with genetic distance. However, for large genetic distances, the differences are reduced, suggesting the existence of more than one cluster of high transmissibility.

### Other HCV outbreaks

The strong association we identified in the AW outbreak between Hamming centrality and transmissibility was further tested using data from nine HCV transmission clusters from the eight other outbreak investigations. We obtained much lower sample sizes than in the AW outbreak here because of the alternate sequencing method used (average number of reads = 50.62). Nonetheless, a strong correlation was observed between Hamming centrality and transmissibility in 7 of the 9 transmission clusters (average *r* = 0.8011, ranging from 0.5650 to 0.947; *p*-value < 0.05).

## Discussion

Stochastic transmission of a limited number of HCV variants from highly heterogeneous intra-host populations generates an opportunity for significant differences in genetic composition of HCV populations found in the source and recipients. Each intra-host HCV population is organized in subpopulations [34] which can be visualized using *k*-step networks [7]. Simulation experiments of transmission events have previously shown that stochastic sampling of a very small fraction of variants from the source results in picking representatives from each large subpopulation [7], indicating that each HCV subpopulation in the recipient has a very high probability to be founded by variants transmitted from the source. However, not all founded subpopulations increase their size at the beginning of infection in a new host to the level detectable by the available sequencing strategies, including UDS used here. Only one or few subpopulations grow in density early in infection, establishing a dominant subpopulation during the acute stage of HCV infection [34]. The other subpopulations become detectable later in infection after decline of the initial HCV variants [34]. Thus, HCV T/F variants from different subpopulations may differ in their biological capacity to rapidly establish a dominant population at the early stages of infection. The frequent finding that the dominant intra-host population in recipients is genetically closest to a minority variants from the source [8] argues against a simple stochasticity of transmission. In addition, identification of common patterns of HCV variants shared among linked individuals strongly suggests specific biological properties for T/F variants [11].

Here, we explored genetic data from one large outbreak involving many cases of HCV infection with epidemiologically confirmed transmission from a single case [16] to identify a differential capacity of the source HCV variants to facilitate establishment of the first dominant population in new hosts. We define transmissibility as a property that can be measured from experimental UDS data and can be applied to other genetically heterogeneous pathogens. This measure of transmissibility is based on: (i) use of average distance (from each sequence in the donor to all sequences in the recipient) instead of minimal distance because the latter created too many ties among sequences which was problematic given the need for ranking; (ii) use of ranks rather than raw distances to control for variation in the range of genetic distances for different recipients; (iii) a compound measure for all recipients was generated using the harmonic mean to reduce probability of producing a high transmissibility value for a single HCV variant located between source subpopulations, variants from which transmitted to different recipients (Fig. 1); (iv) the use of a harmonic mean gives more weight to variants that are very close to one or more recipients; although not used in this study, other schemes such as an expert-based voting system can be applied to generate this compound measure; and (v) use of hamming distance instead of other genetic or patristic distance is justified by the fact, based on our previous finding [24], that hamming distance performs slightly better in identifying closely related individuals linked by transmission.

Transmissibility measured here mitigates several problems associated with the use of shared variants (distance equal 0) rather than all sampled variants at the entire range of genetic distances from each other. For instance, in the AW outbreak, out of the 17 recipients, 5 did not share any variant and 1 shared many variants with the source. This variation in the number of shared variants is most probably associated with:

(i) Stochasticity of sampling: Due to the high level of intra-host HCV genetic heterogeneity, stochasticity of sampling is very high. Even when comparing two samples from the same individual obtained in the same UDS experiment, we found that the average level of variant sharing was approximately 53.80% (average of 576 pairwise comparisons among 24 samples obtained from the same chronically HCV-infected individual, data not shown).
(ii) Sampling times: Sampling from source and recipient cases does not occur at the time of transmission. Owing to the high rate of diversification of RNA viruses, genetic composition of the HCV population sampled weeks or months after the transmission event may not reflect what was present at the time of transmission in the source. In the AW outbreak studied here, all recipients were infected just within 5 weeks and the time between infection and sampling was ~30 weeks.

Both factors have a significant effect on the transmissibility value measured using shared variants. The measure formulated here is robust to variations in sampling, owing to the use of genetic distances from every source variant to all variants from every recipient. Even though the actual T/F variants may not be sampled from recipient because of their low frequency, the genetically proximal HCV variants of sufficient frequency may still be detected and used as a proxy for the T/F variant. The correlation observed here between Hamming distance and the difference in transmissibility among genetically close HCV variants indicates that these variants are similar in their transmissibility, which explains the robustness of our transmissibility measure to sampling stochasticity. In addition, the use of the entire range of distances creates a measure that is continuous rather than binary and enables more accurate modelling.

Although shared variants tend to have a higher average frequency than non-shared variants, there was a very low correlation between the number of recipients who carried a certain source variant, and the variant frequency (*r* = 0.0949; *p* = 0.0464). Transmissibility formulated here did not correlate with the variant frequency. Taken together, these observations suggest that a solely stochastic model accounts for a very small portion of the variance in transmissibility among the source HCV variants. It is conceivable that, to be physically transferred to a recipient, HCV variants must be of sufficient frequency in the source, but other phenotypic traits become important for selecting the variant(s) that establish the first dominant population upon transmission to a new host.

Given the high level of HCV genetic heterogeneity, a large variety of phenotypic traits may be responsible for transmissibility of variants. Depending on the strain and the genetic environments of the host and recipient, different combinations of traits may play a major role in defining each specific transmission event. Identification of common amino acid patterns among shared HCV HVR1 variants in transmission pairs suggests the existence of phenotypic properties associated with transmissibility [11]. However, these patterns were experimentally shown to confer no greater capacity for cell entry than the ones derived from non-transmitted variants [11].

In this study, we found that the Hamming centrality of HCV variants from the source of our outbreak was strongly associated with transmissibility of variants to recipients. This property has the potential to be applied to any highly heterogeneous virus since it is not defined by a motif, but by the location of variants in the sequence space occupied by the entire viral population from the source. A weak association of transmissibility with other measures of network centrality like closeness or betweenness centrality indicates that the Hamming centrality is less dependent than these other measures on comprehensive sampling of HCV variants. The association with the variant centrality in genetic space suggests existence of certain phenotypic traits that can affect transmissibility. One of the possible traits is mutational robustness [35], which has been experimentally observed for HCV [7] and results in generation of numerous viable mutant variants. Another beneficial trait for transmissibility is a weak cross-immunoreactivity with its mutant progeny so that antibodies against T/F variants would be inefficient in neutralizing the genetically proximal HCV variants. In order to play a role in transmission, this weak cross-immunoreactivity should be host independent, which is supported by recent findings regarding the important contribution of cross-immunoreactivity in directing intra-host HCV evolution [36] and limited cross-immunoreactivity among HCV variants in acute infection [37].

The main limitation of this study is that its analysis is based on genetic data from only ten transmission clusters, suggesting that the results should be cautiously applied to other datasets. However, it must be noted that HCV outbreaks are difficult to detect, or fully characterize, because HCV infections are asymptomatic in more than 70% of infected persons for years, which explains limited availability of genetic data from outbreaks. Further improvement of molecular surveillance using novel technologies, like Global Hepatitis Outbreak and Surveillance Technology (GHOST) (Longmire et al. in this issue), is required to develop advanced approaches for accurate tracking of viral transmissions.

## Conclusions

Stochastic sampling does not comprehensively explain genetic differences between intra-host HCV populations found in a source and the recipients. Transmissibility, as formally measured in this study, is associated with certain biological properties that define location of variants in the HCV genetic space. Besides improving accuracy of outbreak investigations, this finding has important implications for understanding of molecular mechanisms contributing to establishment of HCV chronic infection.

## References

[1] Mohd Hanafiah K, Groeger J, Flaxman AD, Wiersma ST (2013). Global epidemiology of hepatitis C virus infection: new estimates of age-specific antibody to HCV seroprevalence. Hepatology.

[2] Alter M (2007). Epidemiology of hepatitis C virus infection. World J Gastroenterol.

[3] Ly KN, Xing J, Klevens RM, Jiles RB, Ward JW, Holmberg SD (2012). The increasing burden of mortality from viral hepatitis in the United States between 1999 and 2007. Ann Intern Med.

[4] Ward JW (2013). The hidden epidemic of hepatitis C virus infection in the United States: occult transmission and burden of disease. Topics Antiviral Med.

[5] Healthcare-Associated Hepatitis B and C Outbreaks Reported to CDC in 2008-2013 [http://www.cdc.gov/hepatitis/Outbreaks/HealthcareHepOutbreakTable.htm].

[6] Domingo E, Sheldon J, Perales C (2012). Viral quasispecies evolution. Microbiol Mol Biol Rev.

[7] Campo DS, Dimitrova Z, Yamasaki L, Skums P, Lau DT, Vaughan G, Forbi JC, Teo CG, Khudyakov Y (2014). Next-generation sequencing reveals large connected networks of intra-host HCV variants. BMC Genomics.

[8] Sagar M (2010). HIV-1 transmission biology: selection and characteristics of infecting viruses. J Infect Dis.

[9] Wang G, Sherrill-Mix S, Chang K, Quince C, Bushman F (2010). Hepatitis C virus transmission bottlenecks analyzed by deep sequencing. J Virol.

[10] Bull RA, Luciani F, McElroy K, Gaudieri S, Pham ST, Chopra A, Cameron B, Maher L, Dore GJ, White PA (2011). Sequential bottlenecks drive viral evolution in early acute hepatitis C virus infection. PLoS Pathog.

[11] D'Arienzo V, Moreau A, D'Alteroche L, Gissot V, Blanchard E, Gaudy-Graffin C, Roch E, Dubois F, Giraudeau B, Plantier JC (2013). Sequence and functional analysis of the envelope glycoproteins of hepatitis C virus variants selectively transmitted to a new host. J Virol.

[12] Li H, Stoddard MB, Wang S, Blair LM, Giorgi EE, Parrish EH, Learn GH, Hraber P, Goepfert PA, Saag MS (2012). Elucidation of hepatitis C virus transmission and early diversification by single genome sequencing. PLoS Pathog.

[13] Preciado MV, Valva P, Escobar-Gutierrez A, Rahal P, Ruiz-Tovar K, Yamasaki L, Vazquez-Chacon C, Martinez-Guarneros A, Carpio-Pedroza JC, Fonseca-Coronado S (2014). Hepatitis C virus molecular evolution: transmission, disease progression and antiviral therapy. World J Gastroenterol.

[14] Kell A, Stoddard M, Li H, Marcotrigiano J, Shaw GM, Gale M (2015). Pathogen-associated molecular pattern recognition of hepatitis C virus transmitted/founder variants by RIG-I is dependent on U-Core length. J Virol.

[15] Astrakhantseva IV, Campo D, Araujo A, Teo C-G, Khudyakov Y, Kamili S: Variation in physicochemical properties of the hypervariable region 1during acute and chronic stages of hepatitis C virus infection. In: Bioinformatics and Biomedicine Workshops (BIBMW), 2011 IEEE International Conference. IEEE Xplore Digital Library; 2011: P. 72 - 78.

[16] Warner AE, Schaefer MK, Patel PR, Drobeniuc J, Xia G, Lin Y, Khudyakov Y, Vonderwahl CW, Miller L, Thompson ND (2015). Outbreak of hepatitis C virus infection associated with narcotics diversion by an hepatitis C virus-infected surgical technician. Am J Infect Control.

[17] Noviello S, Smith P, Chai F, Nainan O, Genovese-Candela A, Armellino D, Farber B, Stricof R, Chang H, Birkhead G (2015). Hepatitis C virus transmission by a cardiac surgeon in the United States.

[18] Chai F, Xia G, Williams I, Rosenberg J, Janowski M, Ginsberg M, Alter M, Nainan O, Gunn R, Janowski M. Transmission of hepatitis C virus at a pain remediation clinic - San Diego, California 2003. In: 43rd annual meeting of the Infectious Diseases Society of America (IDSA): October 6 to 9. Arlington: Infectious Diseases Society of America; 2005. http://www.idsociety.org/Index.aspx.

[19] Lee K, Scoville S, Taylor R, Baum S, Chai F, Bower W, Soltis M, Xia G, Hachey W, Betz T et al. Outbreak of acute hepatitis C virus (HCV) infections of two different genotypes associated with an HCV-infected anesthetist. In: 47th annual meeting of the Infectious Diseases Society of America (IDSA): October 29 to November 1 2009. Arlington: Infectious Diseases Society of America; 2009. http://www.idsociety.org/Index.aspx.

[20] Thompson N, Novak R, White-Comstock M, Xia G, Ganova-Raeva L, Ramachandran S, Khudyakov Y, Bialek S, Williams I (2012). Patient-to-patient hepatitis C virus transmissions associated with infection control breaches in a Hemodialysis unit. J Nephrol Therapeutics.

[21] Fischer GE, Schaefer MK, Labus BJ, Sands L, Rowley P, Azzam IA, Armour P, Khudyakov YE, Lin Y, Xia G (2010). Hepatitis C virus infections from unsafe injection practices at an endoscopy clinic in Las Vegas, Nevada, 2007-2008. Clin Infect Dis.

[22] Moore ZS, Schaefer MK, Hoffmann KK, Thompson SC, Xia GL, Lin Y, Khudyakov Y, Maillard JM, Engel JP, Perz JF (2011). Transmission of hepatitis C virus during myocardial perfusion imaging in an outpatient clinic. Am J Cardiol.

[23] Skums P, Dimitrova Z, Campo DS, Vaughan G, Rossi L, Forbi JC, Yokosawa J, Zelikovsky A, Khudyakov Y (2012). Efficient error correction for next-generation sequencing of viral amplicons. BMC Bioinformatics.

[24] Campo D, Xia G, Dimitrova Z, Lin Y, Ganova-Raeva L, Punkova L, Ramachandran S, Thai H, Sims S, Rytsareva I (2015). Accurate genetic detection of hepatitis C virus transmissions in outbreak settings. J Infect Dis.

[25] Ramachandran S, Xia GL, Ganova-Raeva LM, Nainan OV, Khudyakov Y (2008). End-point limiting-dilution real-time PCR assay for evaluation of hepatitis C virus quasispecies in serum: performance under optimal and suboptimal conditions. J Virol Methods.

[26] Ramachandran S, Zhai X, Thai H, Campo DS, Xia G, Ganova-Raeva LM, Drobeniuc J, Khudyakov YE (2011). Evaluation of intra-host variants of the entire hepatitis B virus genome. PLoS One.

[27] Katoh K, Standley DM (2013). MAFFT multiple sequence alignment software version 7: improvements in performance and usability. Mol Biol Evol.

[28] Quirin A, Cordón O, Guerrero-Bote V, Vargas-Quesada B, Moya-Anegón F: A quick MST-based algorithm to obtain pathfinder networks (∞, n − 1). J Am Soc Inf Sci Technol 2008, 59(12):1912-1924.

[29] Bastian M, Heymann S, Jacomy M. Gephi: an open source software for exploring and manipulating networks. San Jose: International AAAI conference on weblogs and social media; 2009. https://www.aaai.org/.

[30] Latora V, Marchiori M (2001). Efficient behavior of small-world networks. Physics Review Letters.

[31] Freeman L (1977). A set of measures of centrality based on betweenness. Sociometry.

[32] Alvarez-hamelin I, Dall'Asta L, Vespignani A. K-core decomposition: a tool for the visualization of large scale networks. Adv Neural Inf Proces Syst. 2006;18(41):1–13.

[33] Mantel N, Valand R (1970). A technique of nonparametric multivariate analysis. Biometrics.

[34] Ramachandran S, Campo DS, Dimitrova ZE, Xia GL, Purdy MA, Khudyakov YE (2011). Temporal variations in the hepatitis C virus intrahost population during chronic infection. J Virol.

[35] van Nimwegen E, Crutchfield JP, Huynen M (1999). Neutral evolution of mutational robustness. Proc Natl Acad Sci U S A.

[36] Skums P, Bunimovich L, Khudyakov Y (2015). Antigenic cooperation among intrahost HCV variants organized into a complex network of cross-immunoreactivity. Proc Natl Acad Sci U S A.

[37] Campo DS, Dimitrova Z, Yokosawa J, Hoang D, Perez NO, Ramachandran S, Khudyakov Y (2012). Hepatitis C virus antigenic convergence. Sci Rep.

